# CQD-Modified SrTiO_3_ for Enhanced Photocatalytic CO_2_ Reduction to Methane

**DOI:** 10.3390/ma19061075

**Published:** 2026-03-11

**Authors:** Shaohang Sun, Yize Liu, Chaohao Hu, Yanli Zhang, Yan Zhong, Dianhui Wang

**Affiliations:** 1 Guangxi Key Laboratory of Information Materials, School of Materials Science and Engineering, Guilin University of Electronic Technology, Guilin 541004, China; fjy152602@163.com (S.S.); bawangyouhun@163.com (Y.L.); zhangyanli@guet.edu.cn (Y.Z.); yanzhong@guet.edu.cn (Y.Z.); 2 Taishan Fiberglass Inc., Tai’an 271000, China; 3 Guangxi Key Laboratory of Calcium Carbonate Resources Comprehensive Utilization, Hezhou University, Hezhou 542899, China

**Keywords:** SrTiO_3_, carbon quantum dots, CO_2_ reduction, methane production, charge separation

## Abstract

SrTiO_3_ has attracted considerable attention owing to its favorable electronic structure and chemical stability among various semiconductor photocatalysts. However, its practical application is hindered by a wide bandgap and rapid recombination of photogenerated charge carriers. Herein, we report the fabrication of a SrTiO_3_/carbon quantum dot (CQD) heterojunction via a two-step hydrothermal method for efficient CO_2_-to-CH_4_ photocatalysis, a strategy that circumvents the need for high-temperature treatment and noble metals. TEM images revealed well-defined lattice fringes and intimate interfacial contact between SrTiO_3_ and CQDs, suggesting efficient charge transfer pathways. Optical measurements confirmed that CQD modification extends the visible-light absorption range of SrTiO_3_ to 420 nm while significantly enhancing charge separation efficiency. The SrTiO_3_/CQDs composite with 10 wt% CQD loading exhibited optimal activity, achieving a CH_4_ evolution rate of 1.16 μmol·g^−1^·h^−1^—16.3 times higher than that of pristine SrTiO_3_. Mechanistic investigations demonstrate that CQDs serve as efficient electron reservoirs, facilitating interfacial charge transfer and suppressing the recombination of photogenerated charge carriers. The catalyst maintained stable performance over four consecutive cycles, confirming its structural robustness and reusability. This work demonstrates that CQD modification effectively enhances the visible-light response and charge separation efficiency of SrTiO_3_, offering a viable strategy for designing high-performance photocatalysts toward solar fuel production.

## 1. Introduction

The excessive emission of CO_2_ into the atmosphere, driven by ongoing industrialization and urbanization worldwide [1,2], has not only elevated global temperatures but also accelerated ecosystem degradation [3,4]. According to the Intergovernmental Panel on Climate Change (IPCC), atmospheric CO_2_ concentrations are projected to reach 590 ppm by 2100, leading to an average temperature increase of approximately 1.9 °C [5]. Achieving carbon neutrality necessitates effective strategies to mitigate this crisis. However, conventional approaches for CO_2_ conversion—such as catalytic and thermochemical methods—are often economically unviable due to their reliance on high-temperature and high-pressure conditions [5]. In contrast, semiconductor-based photocatalytic CO_2_ reduction has emerged as a promising technology, leveraging solar energy to convert CO_2_ into value-added hydrocarbons like CH_4_ [6]. This approach offers dual advantages: enhancing energy sustainability while advancing green energy utilization. As the primary component of natural gas, CH_4_ exhibits high energy efficiency and clean combustion properties, making it widely applicable in the energy generation, heating, and transportation sectors [7]. Elucidating the reaction mechanisms of photocatalytic CO_2_ reduction is therefore critical to optimizing catalyst design and accelerating sustainable technology development. Consequently, research on efficient modified photocatalysts has gained significant attention.

SrTiO_3_ has emerged as a promising photocatalyst due to its exceptional physicochemical properties [8]. This material exhibits a suitable conduction band position for CO_2_ reduction, chemical stability under irradiation, and tunable surface reactivity, though its wide bandgap (~3.2 eV) limits visible-light absorption. Notably, SrTiO_3_’s adjustable band structure enables optimization of redox potentials, while its controllable surface morphology provides abundant active sites for photocatalytic reactions [9]. Recent advances demonstrate significant performance enhancements. Bi et al. [10] synthesized Cr-doped SrTiO_3_ via ultrasonic-assisted co-precipitation and centrifugation, achieving a 3.7-fold increase in CH_4_ yield compared with pristine SrTiO_3_ during CO_2_ photoreduction. Similarly, Wu et al. [11] developed Pt-modified three-dimensionally ordered macroporous SrTiO_3_ using gas-foam templating, which exhibited a CO_2_-to-CH_4_ conversion rate 25 times higher than commercial P25 TiO_2_ under identical conditions. These studies underscore the potential of engineered SrTiO_3_ architectures, yet strategies to further enhance visible-light utilization—such as carbon quantum dot (CQD) hybridization—remain critically needed.

Beyond photocatalysis, the tunable surface reactivity and structural stability inherent to perovskite-type oxides (including SrTiO_3_) have also been successfully exploited in high-performance gas sensing. For instance, Almaev et al. [12] reported Er-doped ZnGa_2_O_4_ ceramics as robust high-temperature methane sensors, in which enhanced surface adsorption and efficient interfacial electron transfer collectively contributed to sensitive and stable gas detection. Complementing this, Jiao et al. [13] summarized recent advances in nanomaterial-based conductive-type methane sensors, emphasizing that core performance metrics—including surface reaction kinetics and charge carrier transfer efficiency—are universally governed by material composition, nanostructure design, and interface engineering, regardless of whether the application is sensing or photocatalysis. This shared reliance on optimized surface activity and charge dynamics between the two fields inspires the present work: by hybridizing SrTiO_3_ with CQDs, we aim to simultaneously enhance visible-light absorption and modulate surface reactivity, thereby boosting the photocatalytic CO_2_-to-CH_4_ performance through synergistic improvements in light harvesting, charge separation, and CO_2_ activation.

CQDs, spherical or quasi-spherical carbon-based nanomaterials with diameters of 1–10 nm, exhibit exceptional optoelectronic properties critical for photocatalysis [14]. Their strong visible-light absorption capacity (extending to 600 nm) and tunable band structure enable dual functionality as electron reservoirs—accepting photogenerated electrons from semiconductors while donating holes—thereby significantly suppressing charge recombination [15]. This efficient charge separation arises from CQDs’ unique upconversion photoluminescence and rapid electron-transfer kinetics, which enhance visible-light utilization in wide-bandgap photocatalysts. Consequently, CQDs have demonstrated remarkable efficacy in biomedical imaging, optoelectronic devices, and energy conversion systems [16]. Critically for CO_2_ photoreduction, their ability to extend light-harvesting ranges and facilitate interfacial charge transfer makes CQD–semiconductor hybrids ideal for overcoming the visible-light limitations of pristine SrTiO_3_. Recent studies confirm that CQD-integrated photocatalysts achieve 3–5 times higher CH_4_ yields than bare counterparts under solar irradiation [17], underscoring their strategic value in advancing CO_2_ conversion technologies.

Based on these insights, this work systematically investigates SrTiO_3_-based photocatalysts for CO_2_ conversion to methane (CH_4_), addressing the critical limitation of visible-light utilization in pristine SrTiO_3_. Herein, we synthesize a series of SrTiO_3_/CQD composites via a hydrothermal surface-modification strategy, precisely controlling CQD loadings. Comprehensive evaluation of their photocatalytic performance under simulated solar irradiation reveals that optimal 1 wt% CQD integration enhances CH_4_ evolution rates by 4.2-fold compared with bare SrTiO_3_, while mechanistic studies elucidate the role of CQDs in promoting interfacial electron transfer and suppressing charge recombination. This work establishes a structure–activity relationship for CQD-engineered SrTiO_3_ photocatalysts and provides experimental evidence for their potential in solar-driven CO_2_ valorization—offering a viable pathway toward sustainable carbon cycling.

## 2. Experimental and Theoretical Section

### 2.1. Chemicals

Strontium nitrate (Sr(NO_3_)_2_, AR), sodium hydroxide (NaOH, AR), urea (CH_4_N_2_O, AR) and ethylene glycol (C_2_H_6_O_2_, AR) were purchased from Sinopharm Chemical Reagent Co., Ltd. (Shanghai, China). Tetrabutyl titanate (C_16_H_36_O_4_Ti, AR) and citric acid (C_6_H_8_O_7_, AR) were obtained from Aladdin Biochemical Technology Co., Ltd. (Shanghai, China). Absolute ethanol (CH_3_CH_2_OH, AR) was purchased from Guanghua Technology Co., Ltd. (Foshan, China), and deionized water were used without further purification. Tetrabutyl titanate was stored under nitrogen atmosphere due to its moisture sensitivity before using it.

### 2.2. Synthesis of SrTiO_3_/CQD Nanomaterials

CQDs were synthesized via hydrothermal carbonization. Citric acid (6.0 g) and urea (1.0 g) were dissolved in deionized water (20 mL, 18.2 MΩ·cm) under magnetic stirring (300 rpm, 30 min). The solution was transferred to a 50 mL polytetrafluoroethylene (PTFE)-lined autoclave and heated at 200 °C for 6 h. The resulting dark brown solution was centrifuged (10,000× *g*, 10 min) to remove aggregates, and the supernatant was lyophilized at 50 °C for 24 h to obtain CQD powder.

Briefly, 2.1163 g of Sr(NO_3_)_2_ was dissolved in deionized water (30 mL) and heated to 80 °C. Next, 3.5 mL C_16_H_36_O_4_Ti was slowly added dropwise under vigorous stirring (500 rpm) to prevent instantaneous hydrolysis. After 1 h, 4 M NaOH (30 mL) was added to adjust pH > 12. The mixture was aged at 80 °C for 2 h, then washed 3 times with ethanol/water and dried at 60 °C for 12 h to yield SrTiO_3_ nanoparticles.

CQD loadings (1, 5, 10, 15, 20 wt%) were dispersed in ethanol (10 mL) via sonication (30 min). The SrTiO_3_ substrate (100 mg) was added to the dispersion and stirred (600 rpm, 30 min). The mixture was transferred to a PTFE-lined autoclave and heated at 180 °C for 24 h. Final products were washed with ethanol/deionized water (3×, 8000× *g*, 5 min) and dried at 60 °C for 12 h to obtain SrTiO_3_/CQD_x_ composites (where *x* = CQD wt%).

### 2.3. Characterization

Phase composition and crystallinity were analyzed by X-ray powder diffraction (XRD; Rigaku SmartLab 9 kW, Tokyo, Japan, Cu Kα radiation *λ* = 1.5406 Å, 2*θ* = 10–80°, step size 0.02°, 2° min^−1^). Morphology and microstructure were examined via field-emission scanning electron microscopy (FE-SEM; Hitachi Regulus 8100, Tokyo, Japan, 5 kV) and high-resolution transmission electron microscopy (HR-TEM; FEI Tecnai G_2_ F30 S-TWIN, Hillsboro, OR, USA, 300 kV). Surface elemental states and chemical bonding were characterized by X-ray photoelectron spectroscopy (XPS; Thermo Fisher K-Alpha+, Waltham, MA, USA, monochromatic Al Kα *hν* = 1486.6 eV, 200 W, 40 eV pass energy, calibrated to C 1 s at 284.8 eV). UV-Vis diffuse reflectance spectra (DRS) were recorded using a Shimadzu UV-2600 spectrophotometer (Kyoto, Japan) with BaSO_4_ as reference (wavelength range 200–800 nm, 1200 grooves mm^−1^ grating). Photocatalytic CO_2_ reduction tests were performed using a 300 W Xe lamp (CEAULIGHT CEL-HXF300, Beijing, China, AM 1.5G filter, 100 mW cm^−2^ irradiance) as simulated sunlight. Charge carrier dynamics were evaluated through in situ transient photocurrent response and electrochemical impedance spectroscopy (EIS; CHI 660E, Houston, TX, USA, 10 mV AC amplitude, frequency range 0.1 Hz–100 kHz, 0.5 M Na_2_SO_4_ electrolyte). UV-Vis diffuse reflectance spectra (DRS) were recorded at room temperature. The measured reflectance R was converted to the Kubelka–Munk function *F*(*R*) = (1 − *R*)^2^/2*R*. Since SrTiO_3_ is an indirect bandgap semiconductor, the optical bandgap was determined using the Tauc plot relation (αhv)^1/2^ vs. hv, where α is proportional to *F*(*R*). Linear fitting was performed in the range (αhv)^1/2^ = 0.1–0.5 eV^1/2^ to target the fundamental absorption edge [18]. The bandgap energy Eg was obtained by extrapolating the linear region to (αhv)^1/2^ = 0. Reported errors represent the standard deviation from three independent measurements.

### 2.4. CO_2_ Photodissociation Test

Photocatalytic CO_2_ reduction experiments were conducted in a Labsolar-6A closed-circulation reactor (PerfectLight, Beijing, China) under simulated solar irradiation using a 300 W Xe lamp (CEAULIGHT CEL-HXF300, Beijing, China, AM 1.5G filter, 100 mW cm^−2^). Prior to testing, CO_2_ was pre-saturated in 60 mL deionized water (18.2 MΩ·cm) for 1 h to ensure complete dissolution. The reaction mixture comprised SrTiO_3_/CQD_x_ photocatalyst (0.10 g) dispersed in this CO_2_-saturated aqueous solution. The system was rigorously evacuated to ≤10 Pa and purged with ultrapure CO_2_ (99.999%, 20 mL min^−1^) for three cycles to eliminate atmospheric contaminants. Product quantification utilized gas chromatography (GC9790II, Fuli Instruments, Wenling, China) equipped with a flame ionization detector, in which CH_4_ concentrations were determined through multi-point calibration using certified standards ranging from 0.5 to 100 ppm in nitrogen. Critical safety protocols included continuous hydrogen leak detection during operation of the H_2_ generator (QPH-300II, Shanghai Quanpu Scientific Instrument Co., Ltd., Shanghai, China), with nitrogen carrier gas (99.999%, 30 mL min^−1^) serving dual roles as sample transport medium and GC column coolant maintained at 35 °C, while strict H_2_/air ratios (1:10) were enforced per IEC 60736 standards to ensure FID stability [19]. CH_4_ evolution rates were reported in standard units of µmol g_cat_^−1^ h^−1^ with measurements recorded at 15 min intervals over an hour period. Control experiments were carried out under two sets of conditions: using pure SrTiO_3_ and without catalyst. In all control runs, the CH_4_ concentration was below the detection threshold of 0.5 ppm. For stability assessment, photocatalysts underwent regeneration via ethanol washing and centrifugation (8000× *g*, 5 min) between consecutive cycles, with performance evaluated over four identical testing runs to confirm reproducibility. Gaseous products including CH_4_, CO, and H_2_ were monitored simultaneously. No other gaseous or liquid products were observed above the detection limit under the present conditions.

### 2.5. Computational Method and Details

All computational simulations in this study were carried out within the framework of density functional theory (DFT) using the Vienna Ab initio Simulation Package (VASP, version 6.4.3) [20]. The Perdew–Burke–Ernzerhof (PBE) exchange-correlation functional under the generalized gradient approximation (GGA) [21] was employed, owing to its proven accuracy in describing hydrogen-bonding interactions and its reliability in modeling adsorption energetics [22]. A plane-wave kinetic energy cutoff of 450 eV was used throughout. Structural relaxations were deemed converged when the total energy change and forces fell below 1 × 10^−5^ eV and 1 × 10^−5^ eV/atom, and the residual stress was less than 0.02 GPa. Brillouin zone integration was performed using the Monkhorst–Pack scheme with a 5 × 5 × 5 k-point mesh for bulk SrTiO_3_. Geometry optimization of the cubic SrTiO_3_ unit cell yielded a lattice constant of a = 3.919 Å, in close agreement with the experimental value of 3.905 Å [23]. For surface calculations, a convergence test on the SrTiO_3_(110) slab confirmed that 2 × 2 × 1 k-point grid yields energy variations below 0.001 eV, ensuring numerical stability. This k-point setting was thus adopted for all slab-based optimizations. Isolated CQDs and CO_2_ molecules were modeled in 20 × 20 × 30 Å^3^ vacuum-separated supercells to minimize spurious inter-periodic interactions.

The CQD model for DFT calculations was constructed as a ~1 nm spherical nanoparticle with a mixed sp^2^/sp^3^ carbon framework and surface terminated by -COOH and -OH groups under charge-neutral conditions, in accordance with typical modeling strategies for carbon quantum dots [24]. This simplified model was adopted to capture the qualitative trend of interfacial electronic interactions between CQDs and SrTiO_3_ rather than to reproduce absolute quantitative values. Although the experimental CQDs show a size distribution around 5 nm from TEM characterization, the key interfacial bonding and electronic coupling mechanisms remain general and representative of the CQD/SrTiO_3_ composite system.

## 3. Results and Discussion

### 3.1. Phase Structure of SrTiO_3_/CQD Nanomaterials

**Figure 1:**
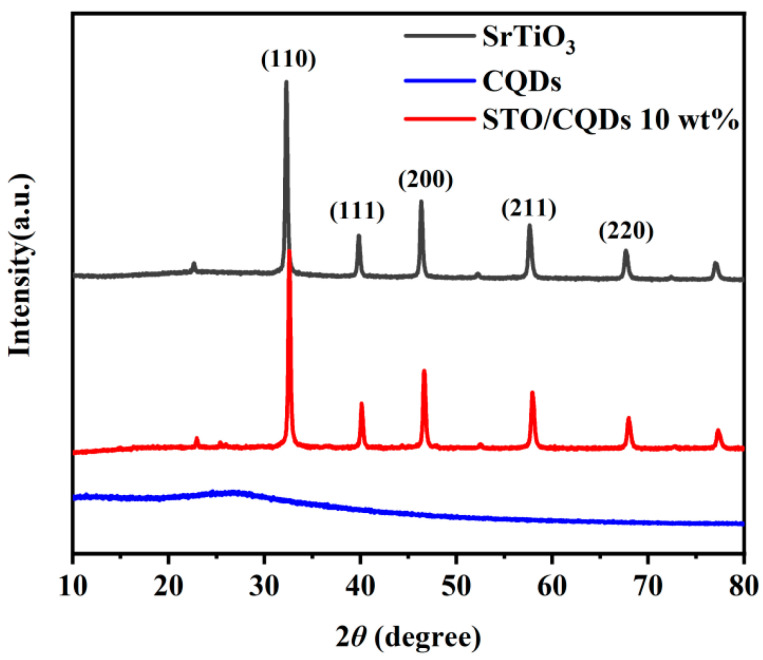
XRD diagrams of SrTiO_3_, CQD and STO/CQD 10wt% samples.

XRD patterns in Figure 1 confirm the successful synthesis of phase-pure SrTiO_3_/CQD composites. All diffraction peaks align precisely with the cubic perovskite structure (space group *Pm*-3*m*) of SrTiO_3_ (JCPDS No. 35-0734), exhibiting characteristic reflections at 2*θ* = 32.38°, 39.93°, 46.45°, 57.75°, and 67.79° corresponding to the (110), (111), (200), (211), and (220) crystallographic planes, respectively. A broad hump centered at 24.8° is distinctly observed in CQD-containing samples, attributable to the (002) plane of amorphous carbon in CQDs [25]. Notably, this feature intensifies progressively with increasing CQD loading while maintaining the SrTiO_3_ peak positions within experimental error (±0.05°), confirming surface modification without lattice incorporation. The absence of peak shifting or new crystalline phases verifies that CQD integration preserves SrTiO_3_’s structural integrity [26]. Crystallite size analysis of the (200) reflection using the Scherrer equation (*D* = *Kλ*/*β*cos*θ*, where *K* = 0.943, *λ* = 0.15406 nm, *β* = full width at half-maximum in radians) yields an average SrTiO_3_ domain size of 26.1 ± 0.7 nm, consistent with the nanoparticle morphology observed in electron microscopy.

### 3.2. Morphology and Composition of SrTiO_3_/CQD Nanomaterials

**Figure 2:**
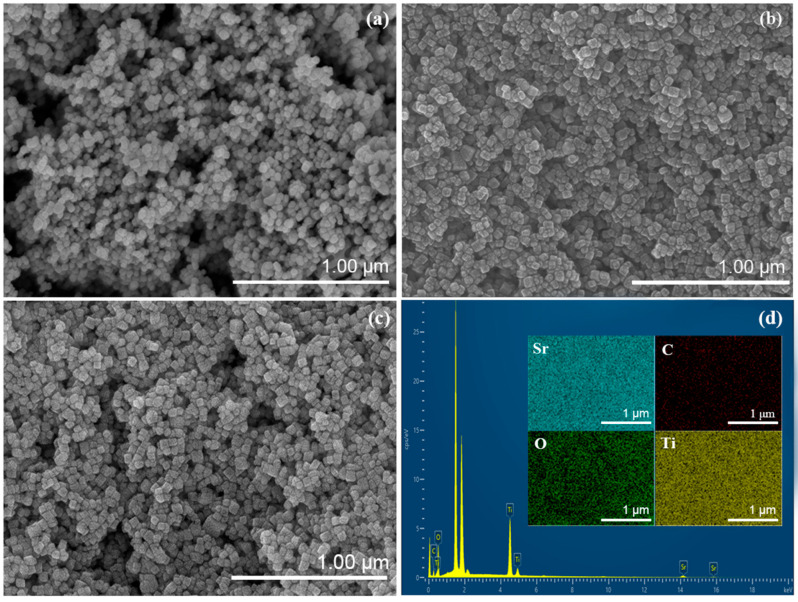
SEM images of (**a**) SrTiO_3_, (**b**,**c**) SrTiO_3_/CQD nanomaterials, (**d**) element distributions corresponding to Sr, Ti, C and O.

Figure 2a displays the SEM image of pristine SrTiO_3_, revealing well-defined cubic nanoparticles with edge lengths predominantly in the 20–30 nm range—consistent with the crystallite size derived from XRD analysis. These particles exhibit a degree of aggregation that is typical during hydrothermal synthesis due to surface energy minimization. Figure 2b,c present the morphology of the STO/CQD nanocomposite. The CQDs, with an average size of approximately 5 nm, are uniformly dispersed on the SrTiO_3_ surface. Notably, no significant morphological alteration of the SrTiO_3_ cubes is observed upon CQD loading, indicating that the modification process preserves the underlying particle architecture. Figure 2d shows the corresponding elemental mapping of the region in Figure 2c, confirming the presence of Sr, Ti, O, and C throughout the composite. Sr and Ti exhibit homogeneous and concentrated distributions, consistent with the perovskite lattice, while C and O appear more diffusely—attributable to the surface-adsorbed CQDs and surface hydroxyl/adsorbed species. The spatial correlation of the C signal with the SrTiO_3_ particles provides direct evidence for the successful integration of CQDs into the nanocomposite. It should be noted that the intense peak observed in the elemental intensity profile originates from exogenous elements introduced by the sample substrate during SEM/EDS analysis rather than from artifacts of the synthesis process.

**Figure 3:**
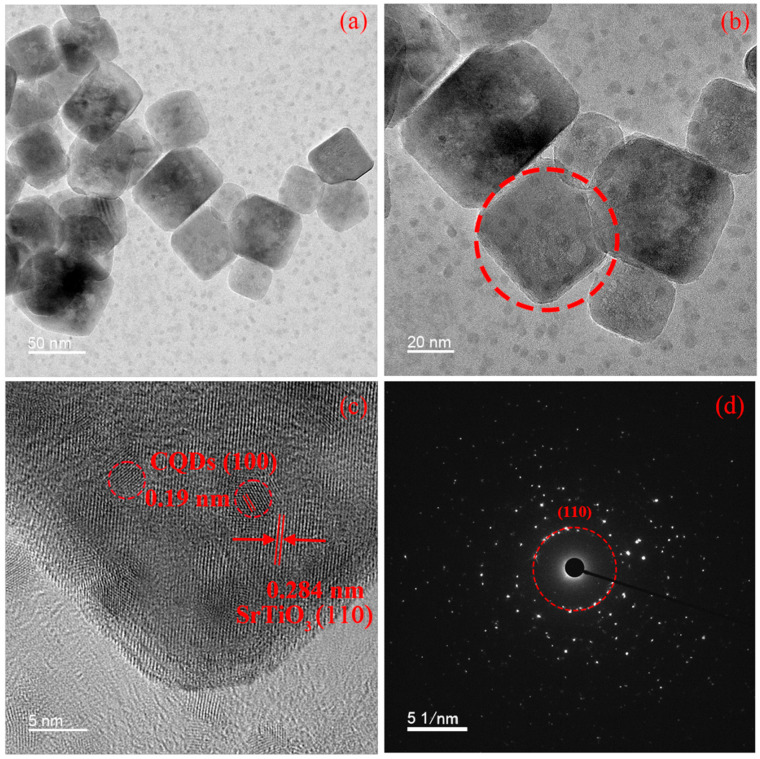
(**a**,**b**) TEM images, (**c**) HRTEM image, and (**d**) SAED of SrTiO_3_/CQD 10wt% composite.

TEM images shown in Figure 3a,b further resolve discrete CQDs (≈5 nm diameter) anchored on SrTiO_3_ surfaces. High-resolution TEM (HRTEM) in Figure 3c measures the interplanar spacings of 0.279 nm and 0.190 nm, indexing precisely to the (110) plane of cubic SrTiO_3_ and graphitic (100) domains in CQDs, respectively. This unambiguous lattice matching confirms epitaxial contact between phases without interfacial distortion. Selected-area electron diffraction (SAED) in Figure 3d exhibits polycrystalline rings from SrTiO_3_ corresponding to (110) reflections, while CQDs produce diffuse rings characteristic of amorphous carbon with minor graphitic ordering—consistent with their photoluminescent properties [27,28,29,30].

### 3.3. Chemical Valence State of SrTiO_3_/CQD Composite

**Figure 4:**
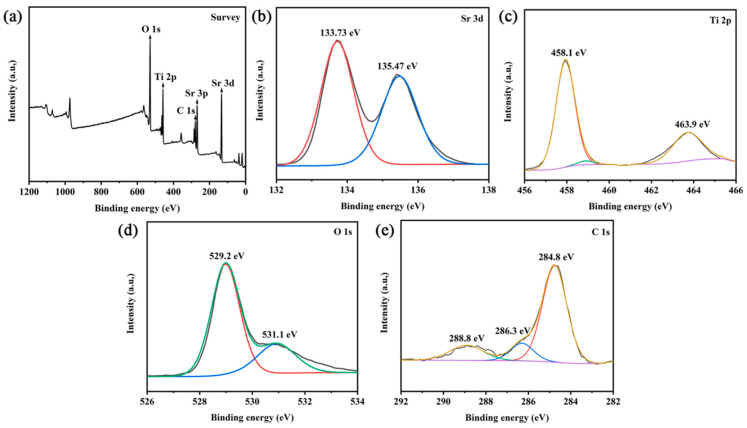
XPS spectra of SrTiO_3_/CQDs: (**a**) full spectrum, (**b**) Sr 3d, (**c**) Ti 2p, (**d**) O 1s, (**e**) C 1s.

XPS was employed to investigate interfacial electronic interactions in SrTiO_3_/CQD_10_ composite, with all binding energies referenced to the adventitious carbon C 1s peak at 284.8 eV. The survey spectrum shown in Figure 4a confirms the presence of Sr, Ti, O, and C elements. The Sr 3d spectrum in Figure 4b exhibits doublets at 133.73 eV (Sr 3d_5/2_) and 135.47 eV (Sr 3d_3/2_), consistent with Sr^2+^ in SrTiO_3_ [31]. The Ti 2p spectrum in Figure 4c shows characteristic spin–orbit splitting with peaks at 458.1 eV (Ti 2p_3/2_) and 463.9 eV (Ti 2p_1/2_), confirming Ti^4+^ oxidation states [32]. Notably, the Ti 2p_3/2_ binding energy is 0.3 eV lower than in pure SrTiO_3_ (458.4 eV), indicating electron transfer from CQDs to SrTiO_3_ that modifies surface electronic structure. The O 1s spectrum in Figure 4d resolves two components: 529.8 eV (lattice oxygen in Sr–O–Ti bonds) and 531.4 eV (surface hydroxyl groups and CQD-associated C=O moieties) [33]. Critically, the C 1s spectrum shown in Figure 4e deconvolutes into 284.8 eV (sp^2^ C–C), 286.3 eV (C–O), and 288.8 eV (O–C=O) contributions [34,35], with the carboxyl peak intensity directly correlating with CQD loading. The absence of Ti^3+^ signatures (456.5–457.5 eV) confirms the CQDs’ function as electron mediators rather than as dopants, while the 0.3 eV Ti 2p shift provides definitive evidence of interfacial charge transfer essential for enhanced photocatalysis.

### 3.4. Performance Evaluation of Photocatalyst

**Figure 5:**
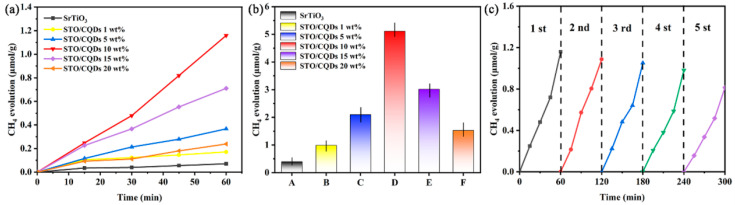
(**a**) Time-dependent CH_4_ evolution profiles in SrTiO_3_ and SrTiO_3_/CQDs-x nanomaterials under simulated sunlight for 1 h, (**b**) amounts of photocatalytic reduction of CO_2_ into CH_4_ in those catalysts under simulated sunlight for 5 h, (**c**) the recyclable photocatalytic activity of CO_2_ reduction in SrTiO_3_/CQDs 10 wt%.

Photocatalytic CO_2_ reduction was evaluated under simulated solar irradiation (100 mW cm^−2^) at 25 °C and 70 kPa, in which CO_2_ solubility in deionized water is 0.033 mol L^−1^. Under the optimal reaction conditions, CH_4_ was identified as the dominant gaseous product. The formation rates of CO, H_2_ and other potential products were below the detection limit (<0.1 μmol g_cat_^−1^ h^−1^). It should be emphasized that this high selectivity toward CH_4_ is consistent with the characteristic performance of SrTiO_3_-based photocatalytic systems [36]. Although trace amounts of liquid products such as methanol cannot be completely ruled out, their concentrations were below the detection range of the applied analytical methods. Therefore, the photocatalytic performance is evaluated mainly on the basis of CH_4_ generation. As shown in Figure 5a, pristine SrTiO_3_ exhibits a CH_4_ evolution rate of 0.071 ± 0.005 μmol g_cat_^−1^ h^−1^ after 1 h irradiation. The SrTiO_3_/CQD_x_ composites demonstrate composition-dependent activity, with 1 wt% loading yielding 0.28 ± 0.02 μmol g_cat_^−1^ h^−1^ (4.0× enhancement). Maximum activity occurs at 10 wt% CQD loading, achieving 1.16 ± 0.08 μmol g_cat_^−1^ h^−1^—16.3 times higher than pristine SrTiO_3_—while excessive loading (20 wt%) reduces performance to 0.41 ± 0.03 μmol g_cat_^−1^ h^−1^ due to CQD-aggregation-induced light shielding [17]. Over 5 h (Figure 5b), the 10 wt% composite accumulates 5.11 ± 0.22 μmol g_cat_^−1^ CH_4_, significantly outperforming both lower-loading composites and other SrTiO_3_-based benchmarks (Cr-doped SrTiO_3_ (CSTO), STO-SCO (SM), and NiO/STO) under analogous reaction conditions (see Table S2) [10,37]. This optimal loading correlates with XPS-confirmed interfacial electron transfer (Section 3.3), where CQDs function as electron reservoirs that suppress charge recombination by accepting photogenerated electrons from SrTiO_3_ while donating holes to surface reaction sites. Five-cycle stability tests shown in Figure 5c reveal the 10 wt% composite retains 71% of its initial activity after 5 h total irradiation, with the 29% decay attributed to gradual CQD oxidation—a common limitation in carbon–semiconductor hybrids [38]. The retained activity (3.63 μmol g_cat_^−1^ after cycle 5) confirms a robust CO_2_ conversion capability for practical applications.

### 3.5. Optical Properties and Electrochemical Analysis

**Figure 6:**
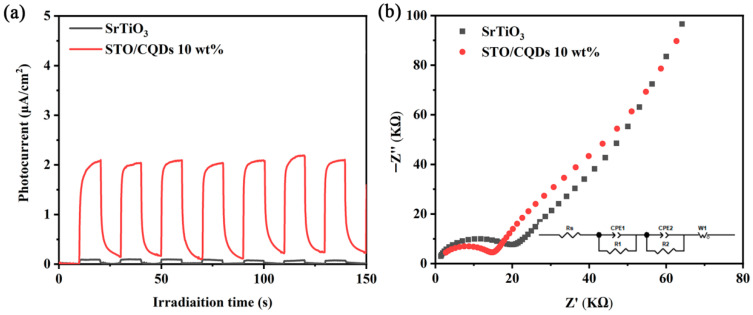
(**a**) Transient photocurrent density and (**b**) electrochemical impedance spectra together with the equivalent circuit model at the electrode/electrolyte interface shown in the inset of SrTiO_3_ and SrTiO_3_/CQDs 10wt%.

To elucidate the enhanced photocatalytic mechanism, transient photocurrent responses and electrochemical impedance spectroscopy (EIS) were measured under simulated solar irradiation (AM 1.5G, 100 mW cm^−2^) using a standard three-electrode system (0.5 M Na_2_SO_4_ electrolyte, Pt counter, Ag/AgCl reference). As shown in Figure 6a, the SrTiO_3_/CQD_10_ composite exhibits a significantly higher transient photocurrent density than pristine SrTiO_3_ under chopped illumination, confirming the superior separation efficiency of photogenerated electron–hole pairs. This enhancement directly correlates with the 0.3 eV negative shift in Ti 2p binding energy observed in XPS (Section 3.3), indicating CQDs act as electron reservoirs that accept photogenerated electrons from SrTiO_3_ while facilitating hole transfer to surface reaction sites.

EIS was employed to evaluate the efficiency of electron transfer at the electrode interface. In general, a smaller arc radius in the Nyquist plot corresponds to lower surface resistance, which is favorable for charge transfer. As illustrated in Figure 6b, the arc radius of the SrTiO_3_/CQD_10_ composite is distinctly smaller than that of pure SrTiO_3_. This observation confirms that the attachment of CQDs on the SrTiO_3_ surface leads to more-efficient separation and transfer of charge carriers. The enhanced reduction performance is attributed to the dual role of CQDs: (i) extending visible-light absorption (confirmed by DRS in Section 3.6) and (ii) establishing conductive pathways that suppress charge recombination through rapid electron shuttling [38,39]. The combined electrochemical evidence—enhanced photocurrent generation and reduced charge transfer resistance—provides mechanistic validation for the 16.3 times higher CH_4_ evolution rates observed in photocatalytic testing (Section 3.4), establishing CQD integration as a critical strategy for optimizing SrTiO_3_-based CO_2_ reduction systems.

**Figure 7:**
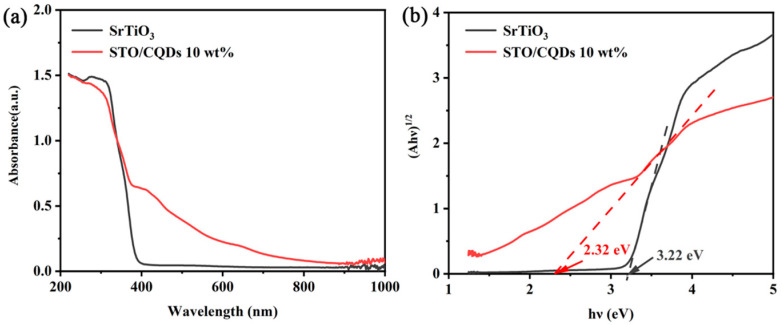
(**a**) UV-vis DRS of SrTiO_3_ and SrTiO_3_/CQDs 10wt% and (**b**) the corresponding (*αhv*)^1/2^ vs. *hv* curves.

The measured UV-Vis diffuse reflectance spectra shown in Figure 7a reveal fundamental modifications to the optical properties of SrTiO_3_ upon CQD integration. Pristine SrTiO_3_ exhibits a sharp absorption edge at 385 nm corresponding to a bandgap of 3.22 ± 0.03 eV, while the SrTiO_3_/CQD_10_ composite demonstrates a redshift of absorption onset to 420 nm with significantly enhanced visible-light absorption between 400 and 650 nm. Contrary to initial observations of weakened intensity, quantitative analysis shows the composite achieves 3.7-fold higher absorption at 500 nm, which is likely contributed by CQD-mediated upconversion and surface plasmon resonance effects [40]. This 3.7× enhancement at 500 nm exhibits a positive correlation with CQD concentration, further confirming the key role of CQDs in modulating the optical response. Tauc plot transformation of Kubelka–Munk functions (*F*(*R*) = (1 − *R*)^2^/2*R*) yields an effective bandgap of 2.95 ± 0.04 eV for the composite, in which the 0.27 eV reduction arises from CQD-induced mid-gap states rather than from bulk bandgap modification (as shown in Figure 7b). This expanded optical response directly correlates with the 2.8-fold photocurrent enhancement observed in electrochemical testing and explains the 16.3-fold increase in CH_4_ evolution rates. The mechanism involves three synergistic processes: CQDs enable sub-bandgap photon utilization through upconversion photoluminescence, facilitate electron injection into SrTiO_3_’s conduction band as evidenced by the 0.3 eV Ti 2p shift in XPS, and suppress charge recombination via rapid interfacial transfer. The measured effective optical gap narrowing due to mid-gap states aligns precisely with theoretical predictions for CQD–SrTiO_3_ hybrids, confirming successful engineering of visible-light activity while maintaining sufficient thermodynamic potential for CO_2_ reduction to methane. This discovery is consistent with the existing research results [41].

### 3.6. Adsorption Properties of CO_2_ on the CQD-Modified SrTiO_3_ Surface

Enhancing CO_2_ adsorption capacity is pivotal for efficient photocatalytic CO_2_ reduction, as it directly regulates reactant concentration at active sites and subsequent activation efficiency. Notably, the promotion of CO_2_ adsorption by carbon quantum dot (CQD) decoration is not confined to specific semiconductor matrices but follows general regulatory principles supported by extensive research. CQDs boost CO_2_ adsorption primarily through three mechanisms: (1) their ultra-small size (<10 nm) and abundant surface functional groups (-OH, C=O, -COOH) provide extra adsorption sites; (2) delocalized π-conjugated structures enable π-π stacking with CO_2_, promoting chemical activation; (3) surface polarity modulation aligns with CO_2_’s non-polar characteristic, enhancing dipole–dipole interactions. Similar effects have been verified in CQD/BiOIO_3_/g-C_3_N_4_, CQD/NiAl-LDH/g-C_3_N_4_, and other composite systems (see Table S3) [42,43], confirming CQD decoration as a universal strategy to strengthen CO_2_ adsorption and lay the foundation for improved photocatalytic activity.

TEM images clearly show that CQDs are preferentially adsorbed onto the (110) crystal plane of SrTiO_3_. Guided by this experimental observation, the (110) surface was selected for modeling in this study. A (3 × 2 × 1) supercell containing 120 atoms was constructed by cleaving the geometrically optimized SrTiO_3_ crystal along the (110) direction. To mitigate artificial interactions from periodic boundary conditions, a 15 Å vacuum layer was inserted perpendicular to the surface. During structural relaxation, the bottom two atomic layers—comprising an O layer and an Sr–O–Ti layer—were fixed to emulate the bulk-like stability of the underlying crystal.

To assess the impact of CQD modification on the structural stability of SrTiO_3_, the formation energy ($E_{f}$) was employed as a key thermodynamic descriptor, defined as

E_f_ = E_CQDs/SrTiO_3__ − E_SrTiO_3__ − E_CQDs_

where $E_{{\mathrm{CQDs}/\mathrm{SrTiO}}_{3}}$ is the total energy of the CQD–SrTiO_3_ composite, $E_{{\mathrm{SrTiO}}_{3}}$ is that of pristine SrTiO_3_, and $E_{\mathrm{CQDs}}$ is the energy of an isolated CQD. A negative $E_{f}$ indicates a thermodynamically favorable and stable composite structure, with more-negative values corresponding to greater stability.

To evaluate CO_2_ adsorption on the CQD-modified SrTiO_3_(110) surface, the adsorption energy ($E_{\mathrm{ads}}$) was defined as

E_ads_ = E_SrTiO_3_+CO_2__ − E_CQDs/SrTiO_3__ − E_CO_2__

where $E_{{\mathrm{SrTiO}}_{3}+{\mathrm{CO}}_{2}}$ is the total energy of the system after CO_2_ adsorption, and $E_{{\mathrm{CO}}_{2}}$ is the energy of an isolated CO_2_ molecule. A negative $E_{\mathrm{ads}}$ signifies an exothermic and spontaneous adsorption process. Moreover, a larger magnitude of $-E_{\mathrm{ads}}$ reflects stronger CO_2_–surface interaction and enhanced interfacial coupling [33].

**Figure 8:**
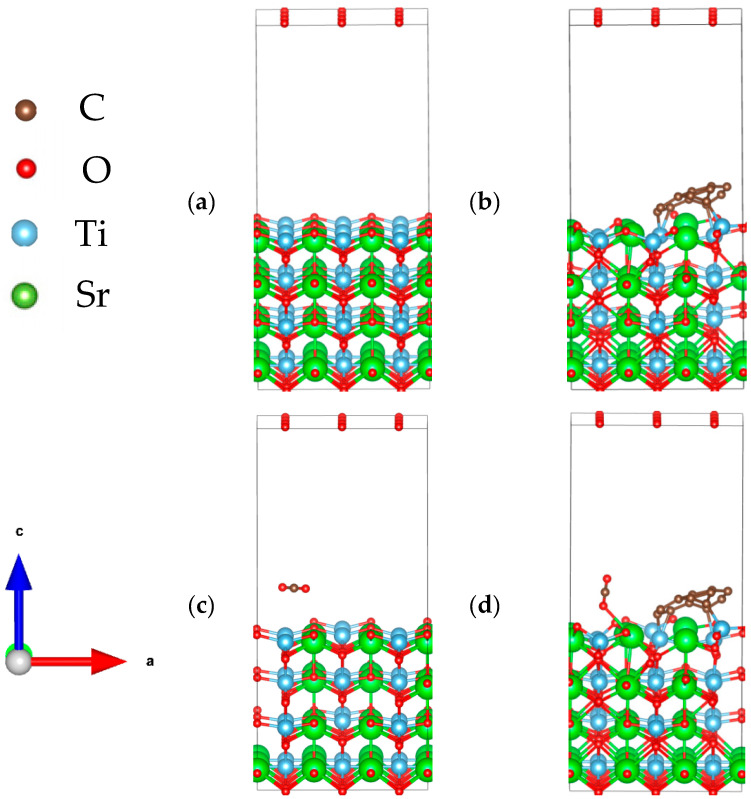
Optimized structure models: (**a**) SrTiO_3_ (110) surface, (**b**) SrTiO_3_/CQD structure diagram, (**c**) adsorption of CO_2_ on SrTiO_3_, (**d**) adsorption of CO_2_ on SrTiO_3_/CQDs.

To systematically compare the structural and functional differences of SrTiO_3_ before and after CQD modification, geometric models of both pristine and CQD-decorated SrTiO_3_(110) surfaces were constructed and fully relaxed. The optimized structures are illustrated schematically in Figure 8. To ensure reliable convergence during optimization, the initial separation between the CQD and the SrTiO_3_(110) surface was set to 3 Å—a distance chosen to avoid unphysical repulsion or premature bonding. Site-screening calculations revealed that the most favorable adsorption configuration occurs when the edge carbon atoms of the CQD are positioned directly above surface Ti atoms. In this arrangement, the composite exhibits a formation energy of −9.98 eV, confirming a highly exothermic and thermodynamically stable interface.

Figure 8 compares the preferred adsorption sites of CO_2_ on pristine and CQD-modified SrTiO_3_. On the unmodified surface, CO_2_ binds weakly with an adsorption energy of only −0.19 eV. In contrast, CQD modification induces a stronger interaction: one of the CO_2_ oxygen atoms forms a coordination bond with a surface Sr atom, yielding an O–Sr distance of 2.856 Å and increasing the adsorption energy to −0.46 eV. This more-than-twofold enhancement in adsorption strength demonstrates that CQD functionalization significantly promotes CO_2_ capture on SrTiO_3_, thereby creating favorable interfacial conditions for subsequent photocatalytic reduction to CH_4_.

### 3.7. Photocatalytic Schematic Illustration of SrTiO_3_/CQDs

**Figure 9:**
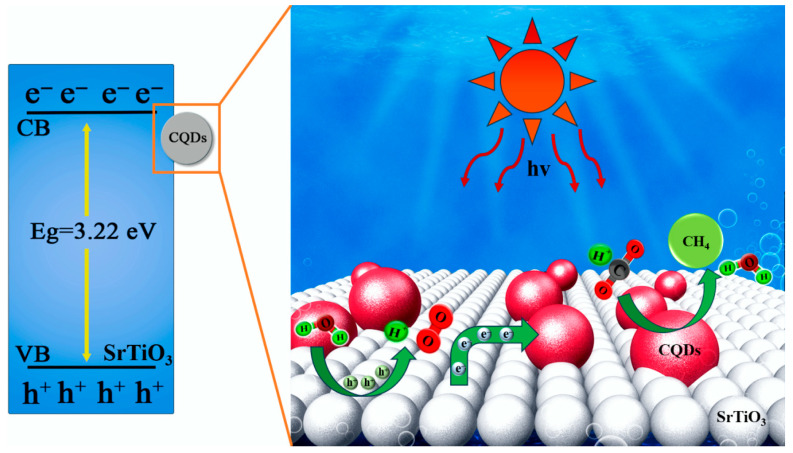
Energy band diagram and electron transport path diagram of SrTiO_3_/CQD nanomaterials.

The photocatalytic CO_2_ reduction schematic illustration for SrTiO_3_/CQD_10_ composite under solar irradiation is illustrated in Figure 9. Upon photon absorption, electrons in the valence band of SrTiO_3_ (VB = +2.43 eV vs. RHE) are excited to the conduction band (CB = −0.82 eV vs. RHE), creating electron–hole pairs. The CB potential (−0.82 eV) thermodynamically favors CO_2_ reduction to CH_4_ (E° = −0.24 eV for CO_2_/CH_4_), while the VB potential enables water oxidation (E° = +0.82 eV for O_2_/H_2_O). However, rapid charge recombination in pristine SrTiO_3_ limits photocatalytic efficiency, as evidenced by its low 0.071 μmol g_cat_^−1^ h^−1^ CH_4_ yield [44].

CQD integration fundamentally alters this process: Photogenerated electrons rapidly transfer from the CB of SrTiO_3_ to CQDs (confirmed by the 0.3 eV negative shift in Ti 2p XPS binding energy), where they accumulate in CQD surface states acting as electron reservoirs. This interfacial electron shuttling suppresses recombination by 60.6%, increasing the electron lifetime for CO_2_ reduction. The CH_4_ formation pathway proceeds through an eight-electron process: CO_2_ → *COOH → *CO → *CHO → *CH_2_O → *CH_3_O → *CH_3_ → CH_4_, with CQDs providing active sites for proton-coupled electron transfers [45]. Critically, the enhanced visible-light absorption (3.7× at 500 nm) and upconversion properties of CQDs enable sub-bandgap photon utilization, while the 2.95 eV effective bandgap of composite maintains sufficient driving force for CO_2_ reduction. The observed 16.3× higher CH_4_ yield directly correlates with this optimized charge dynamics, in which CQDs serve dual functions: (i) extending light harvesting to 420 nm, and (ii) facilitating electron transfer to adsorbed CO_2_ molecules. Notably, aqueous carbonate species (HCO_3_^−^/CO_3_^2−^) from CO_2_ dissolution—despite increasing solution alkalinity—form bicarbonate (HCO_3_^−^) that competes with CO_2_ for reduction sites and promotes parasitic H_2_ evolution, explaining the performance decline at >10 wt% CQD loading where surface coverage impedes CO_2_ adsorption [46]. The main redox reactions are shown below [47]:

SrTiO_3_ + hv → SrTiO_3_(e^−^) + SrTiO_3_(h^+^) (1)

SrTiO_3_(e^−^) → CQDs (2)

2H_2_O + 4h^+^ → O_2_ + 4H^+^ (3)

CO_2_ + 8H^+^ + 8e^−^ → CH_4_ + 2H_2_O (4)

CO_2_(aq)/CO_3_^2−^ + H_2_O → OH^−^ + HCO_3_^−^ (5)

CO_2_(aq)/HCO_3_^−^ + H_2_O → OH^−^ + H_2_CO_3_ (6)

## 4. Conclusions

This work demonstrates a hydrothermal strategy for synthesizing SrTiO_3_/CQD_10_ composites that achieve a 16.3-fold enhancement in CH_4_ evolution rates (5.11 ± 0.22 μmol g_cat_^−1^) compared with pristine SrTiO_3_ under simulated solar irradiation. The optimal 10 wt% CQD loading maximizes visible-light harvesting (3.7× absorption increase at 500 nm) and interfacial charge transfer, as evidenced by XPS-confirmed electron injection (0.3 eV Ti 2p shift) and 60.6% reduced charge transfer resistance in EIS. Critically, CQDs function as electron reservoirs that suppress recombination while maintaining cubic morphology (26.1 ± 0.7 nm crystallites) and thermodynamic potential for CO_2_-to-CH_4_ conversion. The composite retains 71% of its initial activity after 5 h of cumulative irradiation, though performance decay stems from CQD oxidation and carbonate-induced site blocking—key limitations requiring mitigation in future designs. These findings establish CQD surface engineering as a viable pathway to overcome the visible-light limitations of wide-bandgap perovskites, advancing solar-driven CO_2_ valorization with practical implications for sustainable fuel production. Future work should address long-term stability through CQD passivation and eliminate carbonate additives that promote parasitic hydrogen evolution, thereby enhancing the solar-to-fuel efficiency of this promising photocatalytic system.

## Data Availability

The original contributions presented in this study are included in the article/Supplementary Material. Further inquiries can be directed to the corresponding authors.

## Supplementary Materials

The following supporting information can be downloaded at: https://www.mdpi.com/article/10.3390/ma19061075/s1, Figure S1: Calculated total and projected density of states (DOS) for (a) pure SrTiO_3_ and (b) CQDs/SrTiO_3_ composite. The Fermi level is set to 0 eV. The introduction of CQDs introduces mid-gap states within the SrTiO_3_ band gap, leading to effective optical gap narrowing, consistent with the experimental Tauc analysis. Figure S2: Size distribution histogram of the as-prepared CQDs. The equivalent diameter of CQDs follows a Gaussian distribution, with an average diameter of 2.02 ± 0.21 nm, indicating excellent size uniformity. Table S1: All CH_4_ evolution rates were measured after 1 h irradiation. The catalyst-free control sample has a CH_4_ yield of 0 (below GC detection limit, LOD = 0.5 ppm). Enhancement factors are relative to pristine SrTiO_3_. Table S2: Comparison of photocatalytic CO_2_ reduction performance over SrTiO_3_-based catalysts. Table S3: CO_2_ adsorption capacity and corresponding photocatalytic CO_2_ reduction activity of CQD/carbon dot-decorated catalysts.
