# Supplementary material for: CQD-Modified SrTiO3 for Enhanced Photocatalytic CO2 Reduction to Methane
Source: Materials (Basel). 2026 Mar 11;19(6):1075. doi: 10.3390/ma19061075 (PMC13028073; doi:10.3390/ma19061075)
Supplement: Supplementary file 1 [file materials-19-01075-s001.zip › materials-4175613-supplementary.pdf]

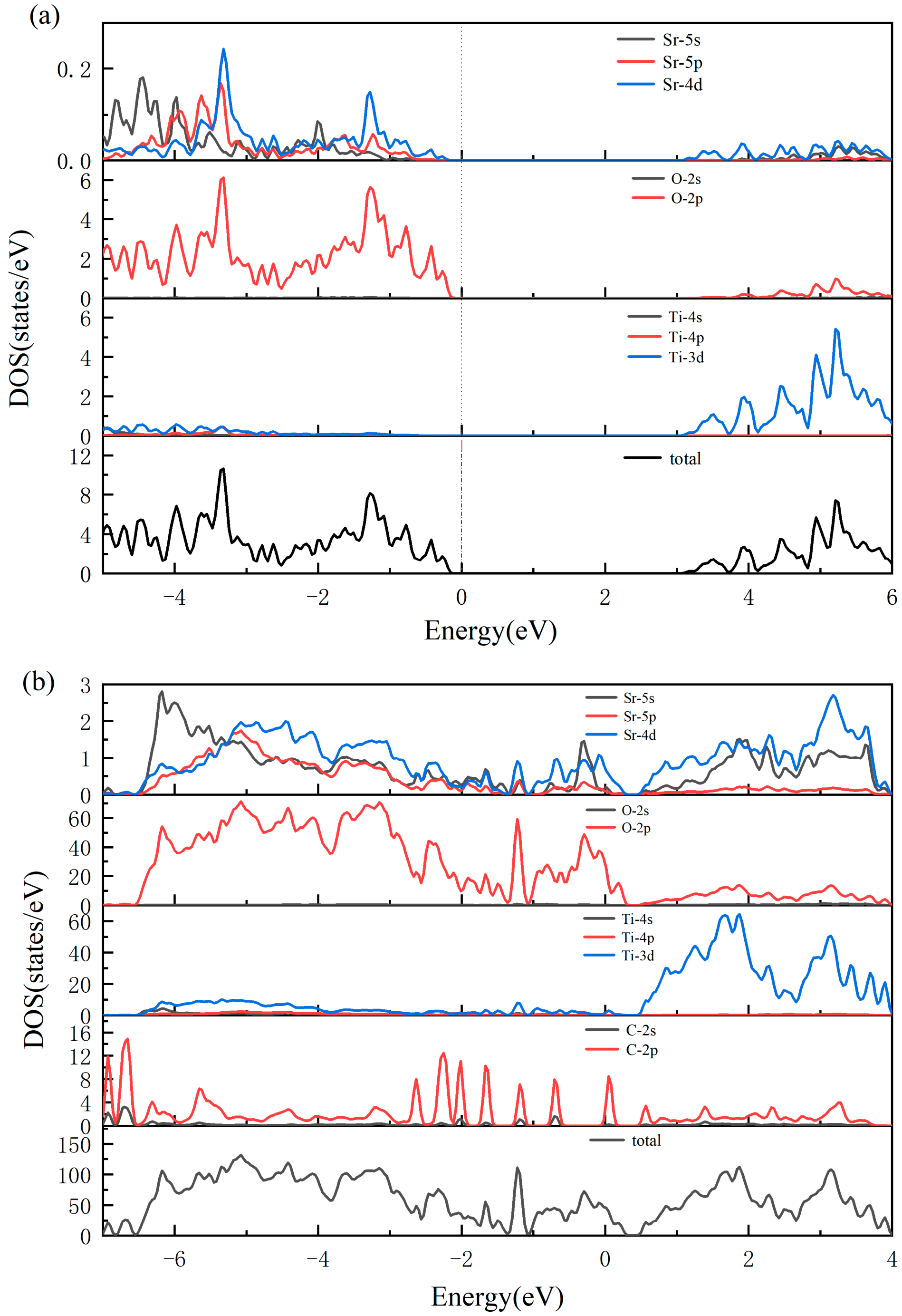

Figure S1. Calculated total and projected density of states (DOS) for (a) pure $SrTiO_3$ and (b) CQDs/$SrTiO_3$ composite. The Fermi level is set to 0 eV. The introduction of CQDs introduces mid-gap states within the $SrTiO_3$ band gap, leading to effective optical gap narrowing, consistent with the experimental Tauc analysis.

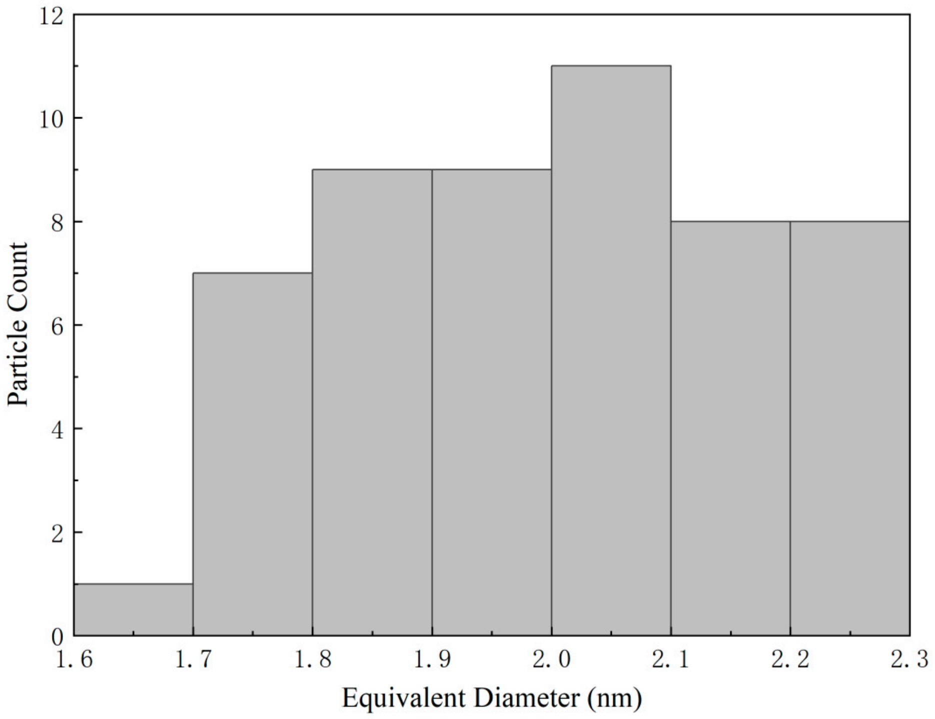

Figure S2. Size distribution histogram of the as-prepared CQDs. The equivalent diameter of CQDs follows a Gaussian distribution, with an average diameter of 2.02 ± 0.21 nm, indicating excellent size uniformity.

Table S1. All CH_4 evolution rates were measured after 1 h irradiation. The catalyst-free control sample has a CH_4 yield of 0 (below GC detection limit, LOD = 0.5 ppm). Enhancement factors are relative to pristine $SrTiO_3$.

**Sample**	**CQD Loading (wt%)**	**CH_4 Evolution Rate (μmol gcat^{-1} h^{-1})**	**Enhancement Factor (vs pristine $SrTiO_3$)**
Pristine $SrTiO_3$	0	0.071 ± 0.005	1.0× (Reference)
$SrTiO_3$/CQD composite	1	0.28 ± 0.02	4.2×
$SrTiO_3$/CQD composite	10	1.16 ± 0.08	16.3×
$SrTiO_3$/CQD composite	20	0.41 ± 0.03	5.8×
**Without catalyst (Control)**	**–**	**0**	**–**

Table S2. Comparison of photocatalytic CO_2 reduction performance over $SrTiO_3$-based catalysts.

Catalyst sample	CH4 generation rate (μ mol gcat-1 h-1)	Enhancement factor vs. pristine $SrTiO_3$	Catalytic features
Pristine $SrTiO_3$	0.071 ± 0.005	1.0×（baseline）	Baseline sample
$SrTiO_3/CQD_x$（1 wt% ）	0.28 ± 0.02	4.0×	Low CQD loading modification
$SrTiO_3/CQD_x$（10 wt% ）	1.16 ± 0.08	16.3×	Optimal performance
$SrTiO_3/CQD_x$（20 wt%）	0.41 ± 0.03	5.8×	Excessive CQD aggregation
Cr-doped $SrTiO_3$（CSTO）	0.88	～12.4×	Cr element doping modification
STO-SCO (SM)	1.029	～14.5×	$SrCO_3$ composite
NiO/STO	～0.726	～10.2×	0.3 wt% NiO

Table S3. CO_2 adsorption capacity and corresponding photocatalytic CO_2 reduction activity of CQD/carbon dot-decorated catalysts

Catalyst Sample	Specific Surface Area ($m^2 \cdot g^{-1}$)	CO_2 Adsorption Capacity ($mmol \cdot g^{-1}$)/Adsorption Efficiency (%)	Main Reduction Product	Catalytic Activity ($\mu mol \cdot g^{-1} \cdot h^{-1}$)	Key Mechanism for Adsorption Enhancement
Pristine $g\text{-}C_3N_4$	21.52	0.032/1.02%	CO, CH_4	CO: 4.0; CH_4: 0.085	Intrinsic triazine ring adsorption
4% CQD/$g\text{-}C_3N_4$	31.81	0.051/1.61%	CO, CH_4	CO: 23.38; CH_4: 20.78	Increased specific surface area + π-π interactions
CQD/OCN-25%	58.7	0.063/1.98%	CH_4	CH_4: 1.12	Defect-induced adsorption + surface functional groups

Catalyst Sample	Specific Surface Area ($m^2 \cdot g^{-1}$)	CO_2 Adsorption Capacity ($mmol \cdot g^{-1}$)/Adsorption Efficiency (%)	Main Reduction Product	Catalytic Activity ($\mu mol \cdot g^{-1} \cdot h^{-1}$)	Key Mechanism for Adsorption Enhancement
Pristine NiAl-LDH/g-C_3N_4	186.4	0.102/3.22%	CO	CO: 1.04	LDH layer Intercalation, adsorption
CQD/NiAl-LDH/g-C_3N_4	685.0	0.172/5.45%	CO	CO: 5.2	CQD-induced pore formation + synergistic adsorption
Pristine $BiOIO_3$/g-C_3N_4	42.3	0.028/0.89%	CO	CO: 32.6	Limited intrinsic adsorption sites
CQD/$BiOIO_3$/g-C_3N_4	117.9	0.075/2.37%	CO	CO: 117.88	CQD-mediated electron accumulation + enhanced adsorption
Lignin-CQD/g-C_3N_4	111.72	0.089/2.82%	CO, CH_4	CO: 19.6; CH_4: 8.3	Abundant surface oxygen-containing groups
